# A matrix-centered view of mass spectrometry platform innovation for volatilome research

**DOI:** 10.3389/fmolb.2024.1421330

**Published:** 2024-10-30

**Authors:** Andras Szeitz, Annika G. Sutton, Steven J. Hallam

**Affiliations:** ^1^ Genome Science and Technology Program, University of British Columbia, Vancouver, BC, Canada; ^2^ Department of Microbiology and Immunology, University of British Columbia, Vancouver, BC, Canada; ^3^ Life Sciences Institute, University of British Columbia, Vancouver, BC, Canada; ^4^ School of Biomedical Engineering, University of British Columbia, Vancouver, BC, Canada; ^5^ Graduate Program in Bioinformatics, University of British Columbia, Vancouver, BC, Canada; ^6^ Department of Biochemistry, Chulalongkorn University, Bangkok, Thailand; ^7^ Bradshaw Research Institute for Minerals and Mining (BRIMM), University of British Columbia, Vancouver, BC, Canada; ^8^ ECOSCOPE Training Program, University of British Columbia, Vancouver, BC, Canada

**Keywords:** volatile organic compounds, selected ion flow tube-mass spectrometry, ion mobility spectrometry-mass spectrometry, proton transfer reaction-mass spectrometry, secondary electrospray ionization-mass spectrometry, time-of-flight mass spectrometry, comprehensive two-dimensional gas chromatography, high resolution multi-reflecting time-of-flight mass spectrometry

## Abstract

Volatile organic compounds (VOCs) are carbon-containing molecules with high vapor pressure and low water solubility that are released from biotic and abiotic matrices. Because they are in the gaseous phase, these compounds tend to remain undetected when using conventional metabolomic profiling methods. Despite this omission, efforts to profile VOCs can provide useful information related to metabolic status and identify potential signaling pathways or toxicological impacts in natural or engineered environments. Over the past several decades mass spectrometry (MS) platform innovation has instigated new opportunities for VOC detection from previously intractable matrices. In parallel, volatilome research linking VOC profiles to other forms of multi-omic information (DNA, RNA, protein, and other metabolites) has gained prominence in resolving genotype/phenotype relationships at different levels of biological organization. This review explores both on-line and off-line methods used in VOC profiling with MS from different matrices. On-line methods involve direct sample injection into the MS platform without any prior compound separation, while off-line methods involve chromatographic separation prior to sample injection and analyte detection. Attention is given to the technical evolution of platforms needed for increasingly resolved VOC profiles, tracing technical progress over time with particular emphasis on emerging microbiome and diagnostic applications.

## 1 Introduction

Volatile organic compounds (VOCs) are gaseous carbon-containing compounds released from biotic and abiotic matrices, manifesting both high vapor pressure and low water solubility (US EPA, 2022). High vapor pressure is correlated with low boiling point and serves as a measure of compound volatility. In some cases, VOCs are associated with adverse health effects depending on concentration and exposure time. The United States Environmental Protection Agency (US EPA) has established a classification system for VOCs that recognizes three primary categories including very volatile organic compounds (VVOCs) < 0°C to 50–100°C, volatile organic compounds (VOCs) 50–100°C to 240–260°C, and semi-volatile organic compounds (SVOCs) 240–260°C to 380–400°C (US EPA, 2022). Volatilome research arises in part from an awareness that both biotic and abiotic matrices emit VOCs, and that VOC profiles obtained using mass spectrometry (MS) platforms can provide useful information about the metabolic status of biological systems as well as potential signaling pathways or toxicological impacts in natural or engineered environments. For example, researchers have identified VOCs in plant (D'Alessandro and Turlings, 2006; Majchrzak et al., 2020), human (Amann et al., 2014; Drabińska et al., 2021; Bauermeister et al., 2022; Ferrandino et al., 2023; Fu et al., 2023), microbial (Boots et al., 2014; Drabińska et al., 2022), food (Cao et al., 2016; Carraturo et al., 2020; Tiwari et al., 2020), and environmental (Liu and Phillips, 1991; Higashikawa et al., 2013; Ullah et al., 2014) matrices. Moreover, several studies have evaluated the role of VOCs in mediating regulatory and metabolic interactions at the population and community levels of biological organization (Audrain et al., 2015; Weisskopf et al., 2021).

In plants, VOCs including terpenoids, fatty acids, and benzenoids are released under normal growth conditions (Dudareva et al., 2004), or in response to environmental stressors such as increased temperature and salinity or herbivory (Sharkey and Yeh, 2001; Mumm et al., 2003; Schröder et al., 2005). Plant-derived VOCs have gained increasing attention in relation to food security and climate change due to potential applications in promoting crop stress responses, pathogen defense, and enhanced biomass production connected to carbon capture and conversion processes (Materić et al., 2015). For example, gas chromatography-mass spectrometry (GC-MS) investigation of *Xanthomonas c. pv. vesicatoria* 85–10 resolved over 50 VOC compounds including several plant growth promoting ketone and methylketone compounds, and one compound linked to growth inhibition of the necrotrophic fungus *Rhizoctonia solani* (Weise et al., 2012). From an environmental perspective, VOCs associated with 48 *Actinobacteria* species isolated from soil and airborne-dust were profiled using GC-MS, resolving 126 predominantly C1 to C5 compounds, including alcohols, ketones, esters with the potential to mediate metabolic interactions among and between microbes and plants (Choudoir et al., 2019). In humans, VOCs have emerged as biomarkers for diagnostic screening and monitoring disease progression (Janssens et al., 2020; Berenguer et al., 2022), as well as detection of pathogens and antimicrobial resistance (AMR) phenotypes (Dixon et al., 2022). For example, VOC detection has been used for biomarker discovery in pulmonary tuberculosis (Fu et al., 2023), cystic fibrosis (Kaeslin et al., 2021), asthma, chronic obstructive pulmonary disease (Ratiu et al., 2020), lung (Ratiu et al., 2020; Temerdashev et al., 2023), prostate (Berenguer et al., 2022) and other type of cancers (Le and Priefer, 2023), urinary tract (Drabińska et al., 2022) and intestinal infections (John et al., 2021), irritable bowel syndrome (Zhang et al., 2023), as well as neurological disorders (Khan et al., 2021). In addition, several studies using isolated *Escherichia coli* have detected VOCs using solid-phase microextraction-gas chromatography coupled with mass spectrometry (SPME-GC-MS) on liquid cultures (Drabińska et al., 2022), or in strains cultivated on blood agar plates using thermal desorption gas chromatography coupled with time-of-flight MS (TD-GC-TOF-MS) (Boots et al., 2014). Similarly, from an industrial perspective, GC-MS analysis of Chinese milk fan (cheese) containing bacteria affiliated with *Lactococcus, Lactobacillus, Raoultella* and fungi affiliated with *Rhodotorula, Torulaspora,* and *Candida* fungi species identified 60 VOCs, including alcohols, aldehydes, ketones, esters, and aromatic compounds contributing to milk fan aroma (Chen et al., 2021).

The emergence of volatilome research is closely coupled with MS platform innovation instigating new opportunities for VOC detection from previously intractable matrices. MS platforms can use either on-line or off-line VOC detection methods that are closely coupled with increasing throughput and resolving power, respectively. On-line methods utilize direct sample introduction to the MS without upstream sample cleanup and compound separation protocols, while off-line techniques employ various analyte separation methodologies prior to MS detection. Previous reviews have discussed the on-line *versus* off-line methods for VOC detection within specific biological systems (Lindinger et al., 1998; Smith and Španěl, 2005; Materić et al., 2015; Ahmed et al., 2017; Lawal et al., 2017; Gould et al., 2021; Westphal et al., 2023). Here we expand on these accounts by presenting a matrix-centered review of volatilome research in relation to platform innovation over time, providing a practical guide for both practitioners and potential end-users with particular emphasis on emerging microbiome and diagnostic applications.

## 2 VOC detection platforms

**FIGURE 1:**
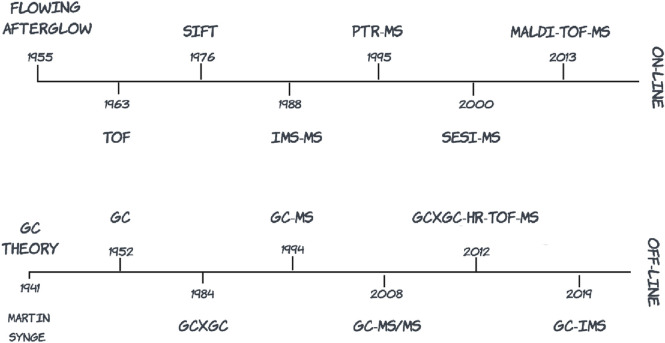
Summary of mass spectrometry platform innovation over the past 60 years in relation to volatile organic compound detection (TOF, time-of-flight; SIFT, selected ion flow tube; IMS-MS, ion mobility spectrometry mass spectrometry; PTR-MS, proton transfer reaction mass spectrometry; SESI-MS, secondary electrospray ionization mass spectrometry; MALDI, matrix-assisted laser desorption ionization; GC, gas chromatography; GCxGC, comprehensive two-dimensional gas chromatography; GC-MS, gas chromatography mass spectrometry; GC-MS/MS, gas chromatography triple quadrupole mass spectrometry; HR-TOF-MS, high resolution time-of-flight mass spectrometry).

Numerous contemporary reviews on analytical methods for detecting VOCs from different matrices are available (Materić et al., 2015; Ahmed et al., 2017; Lough et al., 2017; Lubes and Goodarzi, 2017; Majchrzak et al., 2018; Gould et al., 2021). Figure 1 provides a graphical overview of MS platforms used for VOC detection over time and Table 1 summarizes key scientific literature in relation to different matrix categories and cognate detection platforms. Emphasis is placed on differentiating between on-line methods in which sample preparation is directly coupled to sample injection and analysis, and off-line methods in which the process of sample preparation and analysis are uncoupled. Table 1 includes an overarching selection of MS platform innovation from the early stage of introducing ion molecule reaction MS in 1993 to the latest technological advancements in GCxGC-TOF-MS, in 2023. Review articles are also included in the table to complement research articles with the aim to offer a comprehensive view on VOC analysis combined with MS techniques, in a wide range of matrices.

**TABLE 1:** Summary of analytical techniques and applications used in the analysis of volatile organic compounds with mass spectrometry^*^.

Matrix	Analytical platform	Objectives	Findings	References
Microbes	HS-GC-TOF-MS	Analysis of VOCs in 200 bacterial headspace samples from various species for bacterial identification is reported	Bacterial identification was possible from VOCs including differentiation between a methicillin-resistant and -sensitive *Staphylococcus aureus*	Boots et al. (2014)
	SPME-GC-MS	Monitoring the VOC profiles after adding cephalosporin antibiotics to *Escherichia coli* strains is discussed	Antibiotic susceptibility was detected in urinary tract infection caused by *Escherichia coli* after 2 h	Drabińska et al. (2022)
	SHS-MCC-GC-IMS	An automated approach to differentiate between *Listeria spp*. from VOCs is presented	SHS-MCC-GC-IMS could differentiate between *Listeria* species using their VOC response	Taylor et al. (2017)
	PTR-TOF-MS, SPME-GC-MS	VOC production by *Porphyromonas gingivalis* after treatment with amoxicillin is investigated	Metabolic effects of amoxicillin were reflected in VOCs produced by *Porphyromonas. gingivalis*	Roslund et al. (2022)
	Review	Recent advancements in microbial VOCs and their roles in microbial ecosystems is reviewed in the context of analytical chemistry techniques	Linking the characteristics of microbial VOCs to analytical chemistry techniques could advance the knowledge on volatile-mediated chemical interactions	Weisskopf et al. (2021)
	Review	An overview on the evolution of VOCs and VOC-labeled enzyme substrates to detect pathogenic bacteria is presented	Colorimetric sensor arrays and MS techniques were useful in diagnostics and decision-making, in healthcare and food industries	Lough et al. (2017)
Plants	DI-MS	A review of the applications of DI-MS in real-time plant volatilomics is presented	DI-MS advancements coupled with omics platforms allowed real-time investigation of plant biogenic VOCs	Majchrzak et al. (2020)
	SESI-HRMS	A platform with a broad metabolome coverage for plant VOCs in real-time is described	SESI-MS detected an excess of stress and light induced VOCs emitted by *Begonia semperflorens*	Barrios-Collado et al. (2016)
	GC-MS	Volatiles released by Scots pine twigs after oviposition is analyzed for compounds attracting egg parasitoids	GC-MS analysis showed increased amounts of (E)-β-farnesene that attract egg parasitoids	Mumm et al. (2003)
	MR-GC-DMS	A novel MR-GC-DMS platform using microcontroller boards is reported	MR-GC-DMS could detect VOCs to distinguish between healthy and infected *Rhododendron* plants	Anishchenko et al. (2018)
	HS-SPME-GCxGC-TOF-MS	An aroma comparison in five pear cultivars is investigated	241 volatiles were identified advancing analytical techniques in aroma evaluation	Wang et al. (2019)
	Review	An overview on the role of herbivore-induced plant volatiles (HIPVs) on analytical chemistry methods is discussed	The importance of selecting the appropriate analytical methods could mitigate the challenges in sampling and analyzing HIPVs	D'Alessandro and Turlings (2006)
	Review	A comprehensive guide of analytical methods for plant foliar VOCs, and sampling approaches for plant sciences is presented	The importance of selecting the appropriate sampling methods and analytical techniques for accurate results was emphasized	Materić et al. (2015)
Human Volatilome	Review	An overview of the human volatilome encompassing VOCs in numerous matrices is reported	Comprehensive investigations of VOCs across human matrices contributed to innovative scientific advancements	Amann et al. (2014)
	Review	A review reporting 2,746 VOCs in healthy humans detected mainly with MS techniques is presented	Classified VOCs helped disease diagnosis using the appropriate body matrix for specific research areas	Drabińska et al. (2021)
	Review	An introduction of using untargeted MS techniques to explore human microbiota and its impact on bodily environments	The pivotal role of MS was underlined in mapping microbial functions and physiological impacts	Bauermeister et al. (2022)
Blood	SIFT-MS	SIFT-MS can measure metabolic gases in the headspace of blood culture bottles achieving fast diagnosis in bacteremia or sepsis	Trace gases produced by bacterial cultures were detected after 6 h that was consistent with the gases produced at 24 h	Allardyce et al. (2006b)
	GC-IMS	mVOCs can be analyzed in the headspace of blood culture bottles infected with sepsis-specific pathogens	Using an autosampler with GC-IMS to measure mVOCs could enable point-of-care applications for early detection of sepsis	Drees et al. (2019)
	HS-SPME-GC-MS/MS	A method is developed and validated to quantitate mVOCs as biomarkers in human blood after indoor mold exposure	The method had good linearity, accuracy, precision, limit of detection, and was used to quantify 21 mVOCs in blood	Tabbal et al. (2022b)
	SPME-GC-MS/MS	A method is used to measure BTEX VOCs in human blood at ppt level to conduct PBPK modelling after outdoor and indoor VOC exposure	The method was validated to detect 12 VOCs and applied to analyze samples for a nation-wide biomonitoring study in Canada	Aranda-Rodriguez et al. (2015)
Urine	HS-SPME-GC-MS/MS	A method is developed and validated to quantitate mVOCs as biomarkers in urine after indoor mold exposure	The method had good linearity, accuracy, precision, limit of detection, and was used to quantify 21 mVOCs in urine	Tabbal et al. (2022b)
	Q-MRT-MS	A novel Q-MRT-MS system is introduced featuring an inclined double-orthogonal accelerator and planar gridless ion mirrors with fourth-order energy focusing	Diminishing duty cycles in long flight time instruments could be improved by multiplexing using Encoded Frequent Pushing technology	Cooper-Shepherd et al. (2023)
	PTR-TOF-MS, SIFT-MS	Basic principles and differences between SIFT-MS and PTR-MS are discussed, and real-time breath analysis with the techniques are compared	Real-time analysis of VOCs offered quick results but suffered from interferences. GC separation mitigated interference, but with longer analysis time	Smith et al. (2014)
	PTR-MS PTR-QqQ-MS	PTR-MS can find a marker VOC in the breath of kidney transplant patients that correlates with blood serum creatinine and daily urine production	PTR-TOF-MS and PTR-QqQ-MS confirmed the marker VOC potentially making it possible to monitor kidney functions	Kohl et al. (2013)
Breath	SESI-MS, SESI-HRMS	A review of SESI-MS with an emphasis on quality assurance in data analysis of breath samples in the clinic is reported	SESI-MS was suitable to complement molecular diagnostic methods in early-stage biomarker discovery	Blanco and Vidal-de-Miguel (2021)
	SESI-HRMS	First-time use of SESI-HRMS to find metabolic VOCs in children’s breath with allergic asthma is reported	The technique was useful to identify children with allergic asthma from breath VOCs	Weber et al. (2023b)
	SESI_TOF, PTR-HRMS	First-time systematic evaluation of SESI-HRMS and PTR-HRMS focusing on their suitability to analyze VOCs in adult breath is presented	The sensitive SESI-HRMS found more features but detected less ions in the low mass region than PTR-HRMS.	Bruderer et al. (2020)
	GC-MS	The breath sample analysis of liver cirrhosis patients with Breath Biopsy OMNI Global VOC service and GC-MS to identify candidate biomarkers is reported	VOCs were found as potential biomarkers for progressive liver disease detection that showed good correlation with biomarkers obtained from serum	Ferrandino et al. (2023)
	GC-MS/MS	A needle trap micro-extraction technique with GC-MS/MS is used to analyze VOCs in the breath of patients with heart failure	The optimized method included hydrocarbons, carbonyls, aromatics, sulfur compounds pptv level	Biagini et al. (2017)
	TD-GC-MS	The Peppermint Initiative: A benchmark to establish standardization protocols for VOC analysis in breath is proposed	The Peppermint Consortium encouraged international, cross-platform and interdisciplinary collaboration	Henderson et al. (2020)
	TD-GC-MS/MS	An optimized method is used to analyze carbonyl compounds in the breath of patients with heart failure	The validated method measured aldehydes and ketones at pptv levels to monitor clinical improvement of the patients	Lomonaco et al. (2018)
	TD-GCxGC-TOF-MS	A method is used to investigate whether malarial infection results in characteristic changes of breath profiles in febrile children	A shift was identified in breath composition of Malawian children with six VOCs enabling classification of infection status	Schaber et al. (2018)
	Review	A review of breath biopsy for the measurement of VOCs to monitor respiratory tract, gastrointestinal disorders and sepsis is presented	Online databases for VOCs were built to support breathomics research in patient cohorts with diverse pathologic states	Belizário et al. (2021)
	Review	An overview of sampling and analytical techniques of breath VOCs to diagnose microbial infections and diseases is presented	Microbial infection could produce distinguishable VOCs whose detection could help understand the complex host-pathogen crosstalk	Ahmed et al. (2017)
Food	GC-MS	Development and validation of a GC-MS technique for the analysis of VOCs in individual food and diet samples are reported	The method showed varying concentrations of VOCs in food composites underlining the importance of diet when assessing VOC exposure	Cao et al. (2016)
	HS-SPME-GC-MS	Use of HS-SPME with GC-MS for real-time detection of bacterial contamination of meat is reported	The technique identified unique VOCs supporting an early-warning system for meat contamination	Carraturo et al. (2020)
	Review	Classification of VOCs from horticultural products and linking them with stress factors for quality management are outlined	VOCs as biomarkers could be used to monitor the quality of horticulture goods during storage	Tiwari et al. (2020)
Environment	IMR-MS	An IMR-MS method is used to analyze complex gas mixtures with the advantage of less fragmentation compared to electron-impact ionization	IMR-MS was useful for on-line analysis of car engine exhausts, investigation of catalytic processes and other industrial applications	Lindinger et al. (1993)
	GC-MS/MS	A HS-SPME-GC-MS/MS method is used to measure mVOC biomarkers as occupational health risk agents after indoor mold exposure	The procedure was applied to propose a physiologically based pharmacokinetic model to assess human exposure to indoor mold	Berkane et al. (2023)
	GCxGC-HR-TOF-MS	In diesel fuel samples, advanced data reduction of large datasets obtained with GCxGC-HR-TOF-MS is important for efficient data handling and analysis	Combining Kendrick mass defect and knowledge-based rules, the group type classification reduced complex datasets to a few numerical values	Weggler et al. (2019)
Diagnostics	IMR-MS	The VOC analysis in the headspace of Gram-negative bacterial cultures with IMR-MS as an *in vivo* diagnostic tool is discussed	IMR-MS yielded fast bacterial growth detection and identification offering the possibility of automation	Dolch et al. (2012a)
	SESI-HRMS	Identification of VOC biomarkers in selecting bacterial cultures in cystic fibrosis and assigning molecular structures to features are reported	Several pathogens were distinguished *in vitro* using their VOC profiles and proposed as biomarkers in disease detection in the clinical context	Kaeslin et al. (2021)
	HPPI-TOF-MS	A non-invasive method is implemented to measure VOCs in exhaled breath of TB patients to improve the efficiency of disease diagnosis	The breathomics-based method was accurate, sensitive and specific, offering a simple clinical diagnostic tool for TB screening	Fu et al. (2023)
	Review	MALDI-TOF-MS is described as a method for microbial identification to displace conventional diagnostic techniques	MALDI-TOF-MS would shape and define the clinical microbiology laboratory landscape to accelerate diagnostics and improve patient care	Clark et al. (2013)
	Review	A review of MS techniques with a multiomic approach and integrated science to investigate Enterobacteriaceae phenotypes with carbapenem resistance is reported	Targeted analyses with chromatographic separation and HRMS had potentials to detect molecular signatures and antimicrobial resistance	Dixon et al. (2022)
	Review	VOCs analyzed in headspace of human body fluids with various MS techniques during diagnostic screening and monitoring disease progression in lung cancer are reported	Potential biomarker VOCs in lung cancer showed overlap in human matrices, therefore, standardized trials are needed to validate clinically relevant biosignatures	Janssens et al. (2020)
Multi-omics	GC-MS	Comparative transcriptomics and metabolite profiling are used in tea plants to assess volatile heterosis	Genes and transcription factors with over-dominating expression could serve as candidate genes for breeding high-volatile tea varieties	Zheng et al. (2019)
	GC-MS	Metabolite screening and RNA sequencing of *Magnolia champaca*are employed to discover VOC biosynthesis pathways and floral scent-related genes	GC-MS found 43 VOCs in flowers during sequencing and *de novo*assembly of the transcriptome yielded 47,688 non-redundant unigenes	Dhandapani et al. (2017)
Other analyses	Flowing afterglow	Marking the 50th anniversary of the invention of flowing afterglow with a brief overview of its advancements	Contribution of invertors Eldon Ferguson, Fred Fehsenfeld, Art Schmeltekopf was recognized	Bierbaum (2015)
	SIFT-MS	SIFT-MS is introduced to real-time quantification of trace gases in air and breath. Applications in research areas is reviewed	The technique could detect gases at ppb levels in various matrices. Commercial SIFT-MS instruments were reviewed	Smith and Španěl (2005)
	IMS, IMS-MS	IMS platforms are reviewed with their advantages and disadvantages	IMS interfaced with HRMS could separate and identify unique chemical isomers and isobars	Dodds and Baker (2019)
	IMS-MS	A review of IMS-MS applications to small molecules in drug discovery with limitations and potential applications is presented	IMS-MS, in combination with several analytical techniques, offered structural elucidation in milliseconds	Lapthorn et al. (2013)
	SESI-MS	A SESI model with the effects of several technical parameters on ionization is presented	The ionization mechanism was based on gas phase species rather than charged droplets	Vidal-de-Miguel and Herrero (2012)
	PTR-TOF-MS	A workflow is proposed for the measurement of numerous target compounds in humid air and the results are compared to SIFT-MS data	Using PTR-TOF-MS, less fragmentation and similar information-rich data could be obtained as seen with SIFT-MS.	Romano and Hanna (2018)
	GCxGC-HR MR-TOF-MS FFP EFP	The importance of improving the duty cycle to increase sensitivity of HR MR-TOF-MS FFP systems is discussed	Using Encoded Frequent Pushing spectral multiplexing technique improved the duty cycle in HR MR-TOF-MS FFP systems	Willis et al. (2021)
	PTR-TOF-MS, SPME-GC-TOF-MS	Two MS techniques are employed to monitor the VOC production during 3D printing	Quantitative VOC emissions obtained with PTR-TOF-MS were confirmed with qualitative analysis with SPME-GC-TOF-MS.	Wojnowski et al. (2022)
	TD-GCxGC-TOF-MS, PTR-TOF-MS	VOC emission and uptake of components from the breath sampling device ReCIVA are assessed	Thermal pretreatment of ReCIVA components reduced VOC emissions, and uptake differed for various parts of the device	Pham et al. (2023)
	Review	A review on the analyses of aroma VOCs with advanced MS techniques in food products and chemometrics is presented	In volatile metabolomics, lack of metabolite-specific libraries limited the identification and structural elucidation of VOCs	Lubes and Goodarzi (2017)
	Review	An overview of hyphenated, real-time MS techniques with advantages, limitations and examples is presented	Hyphenated techniques had good qualitative data with long run time. Real-time techniques offered good quantitative data in short analysis time, but only tentative qualitative results	Gould et al. (2021)

For abbreviations, please, refer to Supplementary Material.

### 2.1 On-line methods

On-line methods associated with real-time detection of analytes require minimal preparation, are compatible with both portable and high-throughput platform integration and provide relatively rapid results with reduced price point per sample. However, complex samples containing multiple structurally related analytes present a particular challenge for on-line detection.

#### 2.1.1 Flowing afterglow and SIFT-MS

The flowing afterglow method was developed over 60 years ago to quantitatively measure ion-molecule reaction rate constants. This method utilized a microwave discharge to ionize a primary gas, with the resulting luminous glow migrating to the reaction area, where an ion-molecule reaction transfers charge to neutral species introduced into the buffer gas (Ferguson et al., 1969). In the 1970s flowing afterglow was implemented in a selected ion flow tube (SIFT) where a positively charged single species low energy primary beam was used to ionize neutral components present in a carrier gas, and the reaction was detected using quadrupole MS (Adams and Smith, 1976). The low energy ionization associated with SIFT-MS resolves fewer analyte fragments with less complex mass spectra formation in gas mixtures and alternative precursor ions can be matched with different matrices to obtain representative product ions. For example, SIFT-MS platforms have been used to quantify trace amounts of gas, even in the parts per billion (ppb) range (Smith and Spanel, 1996) down to parts per trillion volume (pptv) (Bierbaum, 2015; Smith et al., 2022) in both air and human breath using primary ion beams composed of dioxygenyl (O_2_^+^), hydronium (H_3_O^+^), or nitrosonium (NO^+^) (Španěl et al., 1999; Smith and Španěl, 2005) ions. SIFT-MS has also been used to monitor bacterial growth (Allardyce et al., 2006a; Allardyce et al., 2006b; Scotter et al., 2006) and to explore ion-molecule reaction kinetics in breath collection bags compared to other methods (Malásková et al., 2019).

#### 2.1.2 IMS-MS

First developed in the 1970s, ion mobility spectrometry (IMS) initially known as plasma chromatography (Cohen and Karasek, 1970), typically uses a radioactive source to ionize analyte gasses that are then separated in a drift tube at atmospheric pressure under an electric field and the counterflow of an inert drift gas (Karasek et al., 1976; Karpas et al., 1988). Due to collisions with the drift gas molecules and electric field acceleration, charged species attain an ion mobility proportional to their shape, charge, size, etc., and their different arrival times at the Faraday plate detector enables selective and sensitive measurements (Hill et al., 1990; Fernández Maestre, 2012). IMS has branched into different forms including drift-tube ion mobility spectrometry, traveling-wave ion mobility spectrometry, trapped ion mobility spectrometry, and field-asymmetric waveform ion mobility spectrometry (Dodds et al., 2020). IMS has been used to measure VOCs in environmental samples (Takaya et al., 2022), and for more complex matrices, IMS can be interfaced with an upstream multicapillary column (MCC) containing up to 1,000 parallel capillaries to separate VOCs prior to ionization (Vautz et al., 2006; Handa et al., 2014). More recent integration of IMS with MS detection (Mukhopadhyay, 2008) enables compound detection based on drift time and mass-to-charge (*m/z*) ratio (Collins and Lee, 2002). Collision cross-section calculations using IMS-MS spectra can also be used to estimate the gas-phase size of ions useful for untargeted analysis (Collins and Lee, 2002; Lapthorn et al., 2013; Dodds and Baker, 2019).

#### 2.1.3 IMR-MS and PTR-MS

In the 1980s ion-molecule reaction mass spectrometry (IMR-MS) emerged as an alternative to SIFT-MS. In contrast to SIFT-MS, IMR-MS employs a two-stage ionization process with Krypton (Kr^+^) or Xeon (Xe^+^) primary beams (Lindinger et al., 1993). During the first stage, primary reagent gas is generated through electron impact ionization, and in the second stage, reagent gas enters a reaction chamber through a lens system where charge is then transferred (Lindinger et al., 1993). Initially, IMR-MS systems were employed to analyze industrial gas mixtures, such as emission from furnaces and motors that required the use of Kr^+^ or Xe^+^ for efficient ionization (Lindinger et al., 1993). In later manifestations, IMR-MS systems capable of alternating between different primary ion beams, including Kr^+^, Xe^+^ or Mercury (Hg^+^) were developed and used to differentiate VOCs produced by Gram-negative and Gram-positive bacteria in headspace analysis of anaerobic blood samples (Dolch et al., 2012a; Dolch et al., 2012b). IMR-MS was also used to analyze VOCs in exhaled breath (Dolch et al., 2015; Meidert et al., 2021).

Proton transfer reaction MS (PTR-MS) introduced in the 1990s is similar to IMR-MS, but uses an ion beam composed of H_3_O^+^ with high proton affinity (Lindinger et al., 1993; Hansel et al., 1995). Use of H_3_O^+^ results in low energy ionization resolving fewer analyte fragments with less complex mass spectra formation in gas mixtures. From a VOC detection standpoint, H_3_O^+^ is effective in detecting trace gas components in exhaled breath (Lindinger et al., 1993; Hansel et al., 1995; Smith and Spanel, 1996). Additionally, the high proton affinity of H_3_O^+^ reduces ion-molecule reactions with major air components including nitrogen (N_2_), oxygen (O_2_), carbon dioxide (CO_2_), and water (H_2_O) (Lagg et al., 1994), reducing potential matrix effects (Bruderer et al., 2020). The singular use of a H_3_O^+^ makes PTR-MS less versatile than SIFT-MS with respect to matching precursor ions with different matrices to obtain representative product ions (Smith and Španěl, 2005). Despite this limitation, PTR-MS has diversified into several forms including PTR-single quadrupole MS (PTR-QMS) (Smith et al., 2014), PTR-triple-quadrupole MS (PTR-QqQ-MS) (Kohl et al., 2013), and PTR-TOF-MS (Smith et al., 2014).

PTR-QMS is a scanning MS platform with a relatively slow data acquisition rate and nominal mass resolution. However, it is effective for targeted analyses to quantitate known VOCs with accuracy and precision (Smith et al., 2014). Combined use of PTR-MS and solid phase microextraction gas chromatography time-of-flight mass spectrometry (SPME-GC-TOF-MS) has been used for monitoring applications (King et al., 2010; Majchrzak et al., 2018) including real-time detection of VOCs released from thermoplastics during 3D printing (Wojnowski et al., 2022). In addition, direct infusion (DI) PTR-MS has been used for real-time detection of VOCs released from plants in combination with multi-omic sequencing to establish a monitoring network to refine global emission budgets and observe plant metabolism at different levels of biological organization (Majchrzak et al., 2020). PTR-QqQ-MS is an extension of PTR-QMS providing higher specificity and sensitivity (Kohl et al., 2013). PTR-TOF-MS platforms collect full mass spectra with high mass resolution (Dodonov et al., 2000; Cooper-Shepherd et al., 2023) making them effective in separating isobaric compounds with high sensitivity and potentially accurate mass for untargeted analyses of complex matrices with many analyte fragments (Smith et al., 2014). Biomarkers from exhaled breath of patients with kidney dysfunction have been identified using a combination of PTR-MS, PTR-TOF-MS with structural elucidation using PTR-QqQ-MS (Kohl et al., 2013).

#### 2.1.4 SESI-MS and Orbitrap-MS

First introduced in the late 1980s, electrospray ionization (ESI) made it efficient to ionize liquid phase polar molecules using a sensitive and low energy process (Whitehouse et al., 1986; Fenn et al., 1989; Fenn, 2002). In a variation of ESI called secondary electrospray ionization (SESI) developed in the 2000s, neutral compounds in gas phase were introduced into the nebulized spray of an ESI stream, where ionized droplets transfer charge to the gaseous species followed by MS detection (Wu et al., 2000; Vidal-de-Miguel and Herrero, 2012). SESI-MS has been used to analyze volatile fatty acids (VFAs) in exhaled breath (Martínez-Lozano et al., 2011) and has been used in combination with high-resolution MS (HRMS) time-of-flight (TOF) and Orbitrap instruments. The first commercial Orbitrap platform was introduced in 2005 for high-resolution mass spectrometry. Produced ions enter a curved trap, where they are collisionally cooled and enriched. The concentrated ion packets are injected orthogonally into the orbitrap where they go into an axial oscillation along a central electrode with a frequency that is proportional to their *m/z* ratio. The central electrode has an opposing electrical charge, and ion stability is achieved by high velocity oscillation that prevents the ions from crashing into the electrode. The resulting resonance signal undergoes a mathematical treatment with Fourier transform, where the oscillation signal is converted to a mass spectrum (Makarov, 2000; Zubarev and Makarov, 2013).

Both SESI and Orbitrap are typically combined with off-line chromatography methods to improve compound identification and mass resolution. SESI-HRMS with TOF has been employed to identify biomarkers for cystic fibrosis in bacterial cultures (Kaeslin et al., 2021), to benchmark PTR-HRMS results (Bruderer et al., 2020), and to monitor VOC production in plants over light-dark cycles (Barrios-Collado et al., 2016). More recently SESI has developed into so-called super SESI, an advanced, electrode-free design with less background noise and memory effects (Blanco and Vidal-de-Miguel, 2021). Super SESI-HRMS with TOF and Orbitrap has been used to profile VOCs in exhaled breath (Blanco and Vidal-de-Miguel, 2021; Weber et al., 2023a; Weber et al., 2023b). Orbitrap-HRMS has also been used to detect VOCs associated with cometary ice analogs (Javelle et al., 2021), as well as SVOCs emanating from environmental dust samples (Pourasil et al., 2022), and plant volatilome (Majchrzak et al., 2020).

#### 2.1.5 TOF-MS

Time-of-flight MS (TOF-MS) introduced in the mid-1950s accelerates ions in an electric field within a flight tube. Ions with a smaller *m/z* ratio travel faster, while those with a higher *m/z* travel slower. Ions are simultaneously detected at a microchannel plate detector, where cascades of electrons are converted into photons amplified by a photomultiplier to generate signals for measurement (Cotter, 1989). The difference between the arrival time of ions at the detector decreases as their *m/z* values increase, and ions with the same *m/z* ratio arrive with a time distribution that can overlap with adjacent ions, reducing mass resolution. Linear TOF-MS instruments, particularly matrix-assisted laser desorption ionization TOF MS (MALDI-TOF-MS) systems are now routinely used in analytical labs with applications as varied as proteomics, biomarker discovery, imaging, materials and environmental monitoring (Clark et al., 2013). A more intricate TOF-MS design is the orthogonal acceleration TOF-MS (oaTOF-MS), where ions are accelerated in the drift region perpendicular to their original direction. Except for the MALDI-TOF-MS, most contemporary TOF-MS platforms use orthogonal acceleration and are commonly referred to as TOF-MS.

High resolution TOF-MS instruments emerged in the 1990s with improved mass resolution using collisional focusing (Douglas and French, 1992) and orthogonal acceleration (Guilhaus et al., 2000), or reflectrons (Mamyrin et al., 1973; Wang et al., 1994; Weggler et al., 2019; Cooper-Shepherd et al., 2023) to increase resolution and the flight path of the ions. A typical drift length in earlier linear oaTOF systems was around 1.5 m (Guilhaus et al., 2000), and this length could be significantly extended by employing different geometric designs. However, increasing the number of passes across mirror grids in reflectrons reduces sensitivity and duty cycle (Cooper-Shepherd et al., 2023). Duty cycle (DuC) describes the proportion of time that ions can enter the TOF for analysis. As mass resolution increases, the duty cycle decreases, with the highest duty cycle attained for the highest *m/z* ion and diminishing for smaller ions (Chernushevich et al., 2017; Willis et al., 2021). The introduction of a linear ion trap/release setup also referred to as “Zeno pulsing” has enabled nearly 100% DuC over a wide *m/z* range using V- (Loboda and Chernushevich, 2009) and W-geometry systems (Chernushevich et al., 2017) in TOF-MS configurations with 20,000 and 90,000 resolving power, respectively. More recently, high resolution, accurate mass, quadrupole-multi-reflecting time-of-flight (Q-MRT) MS instruments featuring open-loop, planar, gridless ion mirrors with fourth-order energy focusing and a 48 m flight path have reached over 200,000 resolving power with sub-ppm mass accuracy (Cooper-Shepherd et al., 2023).

In conventional TOF-MS systems, the push cycle in the accelerator region is followed by a pause period until the largest *m/z* ion arrives at the detector. With longer flight paths, the waiting period may become excessively long, rendering the DuC impractical. Diminishing DuC in instruments with extended flight times can be mitigated by multiplexing using Encoded Frequent Pushing (EFP) technology, where the flight tube is continually filled with ions from subsequent pulses, and the time offset of the pulse frequency is encoded in a sequence that can be accurately decoded to represent individual signals in Q-MRT systems (Willis et al., 2021; Cooper-Shepherd et al., 2023). For example, high resolution multi-reflecting time-of-flight mass spectrometry with folded 20 m flight path (HR-MR-TOF-MS FFP) combined with EFP technology reaches a 50,000 resolving power. Analysis of Egyptian mummy bandage extracts using a GCxGC-HR-MR-TOF-MS FFP with EFP system indicated improvements in signal intensity, dynamic mass range, accurate mass data, and limit of detection confirming the reliable operation of decoding algorithms and hardware with increased transient length in HR-TOF-MS instruments (Willis et al., 2021; LECO Corporation White paper, 2021).

TOF-MS can be combined with numerous off-line methods for VOC detection. For instance, a PTR-TOF-MS method was developed for improved detection of aldehydes, fatty acids, and phenols in humid air (Romano and Hanna, 2018). The same method was used to profile VOCs produced by *Porphyromonas gingivalis*, a common constituent of the human oral microbiome identifying biomarkers in exhaled breath and saliva samples (Roslund et al., 2022). Advances in high-speed electronics, collisional cooling and orthogonal acceleration have increased the resolution of TOF instruments (Douglas and French, 1992; Guilhaus et al., 2000; Chernushevich and Thomson, 2004). In such instruments, ions pass through a multi-pole lens system filled with a low-pressure inert gas and applied radio frequency (RF) resulting in collisional cooling that forms a narrow and dense ion beam. The beam enters an acceleration region, where it is further enriched and the ions are pushed as concentrated packets into the flight tube in an orthogonal direction (Dodonov et al., 1987; Dodonov et al., 1993; Dodonov et al., 2000). High resolution TOF-MS (HR-TOF-MS) has been used to profile VOCs in exhaled breath (Weber et al., 2023a; Weber et al., 2023b), to identify biomarkers for cystic fibrosis in bacterial cultures (Kaeslin et al., 2021), and to benchmark SESI and PTR-HRMS results (Bruderer et al., 2020).

### 2.2 Off-line methods

Off-line or hyphenated methods integrate chromatographic separation processes upstream of analyte detection. For example, gas chromatography to separate sample components prior to MS can improve resolution, allowing for more precise identification and quantification of the analytes. Gas chromatography (GC), formerly known as gas-liquid chromatography, evolved from liquid chromatography (LC) in 1941, when Martin and Synge proposed during their research on liquid-liquid partition chromatography that the mobile liquid phase could be replaced with a gas phase (Martin and Synge, 1941). The first application with GC was published in 1952 reporting the separation of VFAs from other acidic components and it can be considered the first publication on VOC analysis using GC (James and Martin, 1952). Nowadays, VOC profiling involves various types of GC utilizing open tubular capillary columns with diverse stationary phases connected to a wide range of detectors. These methods require more intensive sample preparation and more time to implement with respect to method development and sample analysis. Although the process of sample preparation can be semi-automated, the throughput of off-line methods tends to be much lower than on-line methods. The primary advantage of using off-line methods is the increased resolution of analytes with less deconvolution needed to interpret mass spectra resulting in improved identification within more complex matrices.

#### 2.2.1 GC-MS

First introduced in the 1950s, GC-MS instruments employed packed columns connected to a magnetic sector mass spectrometer (Grayson, 2016). The first GC with a single quadrupole MS (QMS) became commercially available in 1961 (Finnigan, 2016) and has become one of the most widely used platforms for small molecule detection in liquid or gaseous phase samples across diverse matrices. Currently, most contemporary GC-MS platforms use GCs with capillary columns coupled with a QMS and are commonly referred to as GC-MS. GC-MS instruments are recognized for their robustness, user-friendly configurations, affordability, and high chromatographic resolution with low mass resolution, enabling detection and quantitation of analytes with nominal mass (Rey-Stolle et al., 2022).

Liquid samples can be introduced directly into the injection port, where they are vaporized, focused, and carried to the column by the carrier gas in either a split or splitless injection mode. Gas-phase samples are injected in a similar way except the analytes are captured from the headspace (HS) of the sample container using static HS (SHS), dynamic HS (DHS), SPME, thermal desorption (TD), purge-trap, needle trap, etc., methods (Sugita and Sato, 2021) followed by sample introduction to the injection port. Thermally labile compounds, instead of being vaporized, are injected via cool on-column injection. Analytes are separated based on their physico-chemical properties in relation to the column’s stationary phase, carrier gas, oven temperature programming, and other experimental parameters. Analytes eluted from the column are ionized, typically using electron impact (EI) ionization, where an electron beam emitted by a tungsten filament bombards the molecules, fragmenting them into smaller pieces. This process produces radical cations where the molecular ion is typically not observed. While hard ionization EI is useful for quantitation in targeted analysis, it is less optimal when conducting compound characterization, structural elucidation, or untargeted analysis. Alternatively, analytes can be ionized with chemical ionization, where the electron beam ionizes a reagent gas (e.g., methane, ammonia, etc.) first, then the ionized reagent gas transfers charge to the analyte in a soft ionization process that produces negatively or positively charged ions with the molecular ion and less fragmentation (González et al., 1995; Lubes and Goodarzi, 2017). The ionized species enter a single quadrupole MS, where a constant ratio of direct current (DC)/RF is applied and ramped on the rods of the quadrupole, resulting in ions following either an unstable or stable trajectory. The trajectory of an ion is determined by its *m/z* ratio with unstable ions hitting the rods, discharging, and venting from the system, while stable ions travel through the quadrupole, generating a signal at the detector (Pedder, 2001).

From a VOC detection standpoint, TD-GC-MS has been used to develop standards for exhaled breath (Henderson et al., 2020), and HS-SPME-GC-MS has been used to identify biomarkers of meat spoilage associated with *Salmonella Typhimurium* and *Campylobacter jejuni* reducing the time required for regulatory compliance (Carraturo et al., 2020). Similarly, HS-SPME-GC-MS has been used to profile metabolites associated with the *Candida spp*. volatilome identifying sesquiterpene indicators for *Candida albicans* infection (Fitzgerald et al., 2022). In special cases, GC ionization has been achieved using ESI where the column effluent was introduced into an electrospray plume using a multiple channel ESI MS technique. This method was applied to study the chemical reactions of VOCs with solid catalysts, such as the dehydration of dimethylhydrazine in the presence of mercury oxide (Lee and Shiea, 1998). More recently, GC-IMS has been developed to analyze VOCs in the headspace of both aerobic and anaerobic human blood culture samples, identifying species-specific volatiles that enabled the identification of bacterial strains in bloodstream infections after a 6-h incubations (Drees et al., 2019). Similar to TD-GC-MS, HS-GC-IMS has been used to benchmark VOC detection standards for exhaled breath (Ruszkiewicz et al., 2022) and to profile clinical samples including HS-MCC-GC-IMS system used to detect VOCs associated with *Listeria* spp. infections (Taylor et al., 2017). Anishchenko and colleagues developed a modular and reconfigurable system combined with differential mobility spectrometry, a variant form of IMS, to detect VOCs associated with *Phytophthora ramorum*, a protistan plant pathogen (Anishchenko et al., 2018). Combining GC-MS methods with various collection and detection modules provides a versatile and customizable framework for detecting VOCs from diverse matrices.

#### 2.2.2 GC-MS/MS

The first commercially available tandem GC-MS/MS platform appeared on the market in 2008 incorporating a primary quadrupole (MS1), collision cell, and secondary quadrupole (MS2) with ion optics and focusing lenses. Precursor ions exiting MS1 undergo fragmentation in the collision cell by colliding with an inert, pressurized collision gas (such as helium or nitrogen), and the resulting product ions enter MS2, where they are filtered by applying and ramping a constant DC/RF ratio on the quadrupole rods, as previously explained. The primary advantage of GC-MS/MS lies in its selective and sensitive quantification of targeted analytes in complex matrices, achieved through a multiple reaction monitoring (MRM) mode of operation. In MRM mode, the most abundant precursor and product ions obtained from MS1 and MS2 are paired into a joint MRM signal enabling the targeted detection of the compound with high selectivity and sensitivity. In MRM, unlike ramping voltages, specific DC/RF ratios are applied to MS1 and MS2, ensuring stable trajectories for the precursor and product ions of selected analytes while reducing matrix effects. High-speed electronics enable the rapid interchange of many DC/RF ratios on the quadrupoles in microseconds, allowing simultaneous detection and quantification of numerous analytes in complex matrices (Lubes and Goodarzi, 2017). For example, HS-SPME-GC-MS/MS was used to profile microbial VOCs in human urine and blood as potential biomarkers for indoor mold exposure, resolving 21 analytes as potential occupational health risks (Tabbal et al., 2022b). A modified version of this method was employed to determine urine/air, blood/air, and plasma/air partition coefficients of microbial VOCs in relation to indoor mold exposure (Berkane et al., 2023). In a related study conducted by the Canadian Government, SPME-GC-MS/MS was used to monitor volatile halogenated and BTEX compounds in human blood to determine differences between indoor and outdoor exposure risks (Aranda-Rodriguez et al., 2015). A similar method using HS-GC-MS/MS profiled BTEX and other VOCs in sewage sludge samples from various wastewater treatment plants to better constrain odor control management practices (Byliński et al., 2019). From a biomarker discovery perspective, triple-bed needle trap micro-extraction with GC-MS/MS was used to profile VOCs in exhaled breath of patients with congestive heart failure to identify indicators of disease progression (Biagini et al., 2017; Bellagambi et al., 2021), while the use of a derivatizing agent such as pentafluorobenzyl hydroxylamine pre-loaded on Tenax GR sorbent tubes has been used in conjunction with TD-GC-MS/MS analysis to profile VOCs in exhaled breath from similar patients to monitor clinical improvement over time (Lomonaco et al., 2018).

#### 2.2.3 Multi-dimensional GC (2DGC, GCxGC)

In complex matrices, analytes may persistently coelute even after chromatographic separation using long columns with advanced column chemistry. Efficiently separating multiple coeluting peaks and achieving positive compound identification using deconvolution algorithms can be difficult, especially when working with poorly characterized sample types. Multi-dimensional GC attempts to address these challenges by separating analytes across different phases (Seeley and Seeley, 2013). For example, in two-dimensional GC (2DGC) effluent from the first-dimension column is diverted onto a second-dimension column with a different stationary phase for added separation. The two columns have separate detectors, generating distinct data files. Another option is comprehensive two-dimensional GC (GCxGC) that employs two ovens and columns featuring different stationary phases, along with a modulator for peak manipulation and a single detector (Giddings, 1984; Liu and Phillips, 1991). Modulation is achieved using rapid flow or thermal separation methods to improve analyte detection (Amaral et al., 2020), e.g., thermal modulation using GCxGC with dual-stage quad-jet thermal modulation and cryogenic cooling. During thermal modulation, coeluting peaks arriving from the first-dimension column undergo added separation by entering the modulator, where the peaks are segmented into slices, focused, injected, and subsequently separated in the second-dimension column, where the sliced sections of the peaks represent analytes resolved from previously coeluting peaks in the first dimension (Sarbach et al., 2013). TD-GCxGC-TOF-MS and PTR-TOF-MS have been used to establish standards for profiling VOCs in exhaled breath using the ReCIVA breath biopsy device including assessment of analyte loss resulting and false positive detection (Pham et al., 2023). Similarly, TD-GCxGC-TOF-MS was used to profile VOCs from the exhaled breath of febrile children infected with the malaria causing parasite *Plasmodium falciparum* resulting in the identification of six potential biomarkers associated with infection status (Schaber et al., 2018). Interestingly, these VOCS were related to terpenes known to attract mosquito vectors involved in malaria transmission. From an industrial perspective, tri-bed SPME-GCxGC-TOF-MS has been used to identify 241 VOCs including esters, alcohols, aldehydes, and alkenes involved in determining pear aroma that could be correlated with genetic differences between cultivars (Wang et al., 2019).

## 3 Conclusion

**FIGURE 2:**
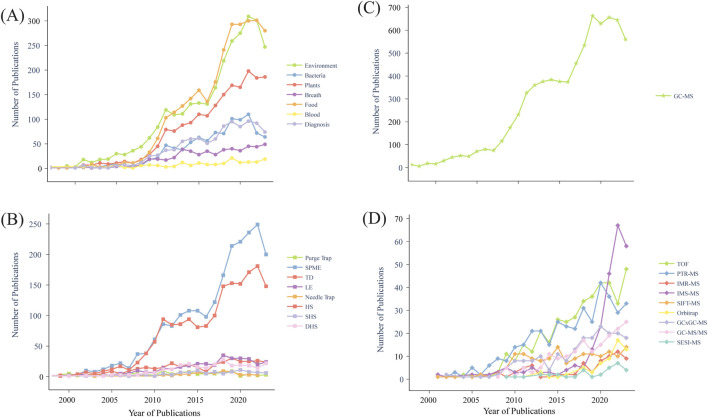
Increasing number of publications related to volatile organic compound detection over the past 20 years for different matrices, methods, and platforms based on PubMed searches. **(A)** various matrices, **(B)** extraction methods (SPME, solid phase microextraction; TD, thermal desorption; LE, liquid extraction; HS, headspace; SHS, static headspace; DHS, dynamic headspace), **(C)** GC-MS (gas chromatography mass spectrometry), **(D)** other mass spectrometry (MS) platforms (TOF, time-of-flight; PTR-MS, proton transfer reaction MS; IMR-MS, ion molecule reaction MS; IMS-MS, ion mobility spectrometry MS; SIFT-MS, selected ion flow tube MS; GCxGC-MS, comprehensive two-dimensional gas chromatography MS; GC-MS/MS, gas chromatography triple quadrupole MS; SESI-MS, secondary electrospray ionization MS).

This review explores both on-line and off-line methods used in VOC profiling with MS from different matrices. Attention is given to the technical evolution of on-line and off-line methods needed for increasingly resolved VOC profiles, tracing technical progress over time with particular emphasis on emerging microbiome and diagnostic applications (summarized in Table 2). VOC profiling has grown expansively over the past 2 decades across multiple different platforms. It is expected that this trend will continue as more scientists and clinicians turn to increasingly sensitive MS detection platforms including different forms of GCxGC-TOF-MS and PTR-TOF-MS for development of diagnostic and monitoring solutions. At the same time emerging bioinformatics workflows enabling integration of multi-omic data sets (DNA, RNA, proteins, metabolites) promise to invigorate and inform volatilome research across increasingly diverse matrices (Figure 2). For example, Guo and colleagues developed an automated cuvette system in conjunction with on-line PTR-ToF-MS and off-line GC-MS to evaluate fungal VOCs from 43 individual fungal isolates and used the resulting spectra to identify patterns of covariation that informed a machine learning model for biomarker detection within higher level taxonomic groups or functional guilds (Guo et al., 2021).

**TABLE 2:** Summary of advantages and disadvantages of on-line and off-line mass spectrometry techniques^*^.

MS platform	Analytical technique	Advantages	Disadvantages	References
On-line	Flowing afterglow	Pioneering technology for the quantitative measurement of ion reaction constants between charged and neutral species, leading to the development of SIFT-MS.	Charge transfer reactions of positive ions to neutrals can be measured more efficiently in certain cases with more direct methodologies	Ferguson et al. (1969)
	SIFT-MS	Easy to operate and maintain. No sample preparation. Results in minutes. Thermal ionization yields less fragmentation and simple mass spectra. Switching between reagent ions (i.e., O_2_^+^, H_3_O^+^, NO^+^) can be optimized for best analyte detection. Sensitive and more selective than PRT-MS.	Cannot distinguish between isobars. Quantitation is possible only after calibration with known analytes. Bulky pumping systems are required to maintain separate vacuum regions of the instrument. Requires high purity helium or hydrogen as carrier gas	Prince et al. (2010)
	IMS-MS	Fast, portable, can detect trace amounts of analytes. Separation is based on collisional cross section and separates isomers. High throughput. Suitable for field applications, such as environmental, food, or homeland security samples. Result available in minutes	Sensitive to temperature and humidity changes. Compound resolution efficiency decreases with temperature and humidity that degrade samples. Not suitable for non-volatile analytes. Complex mass spectra, therefore, off-line techniques may be needed to confirm results	Lapthorn et al. (2013)
	IMR-MS	Low-energy chemical ionization. Binary collision process. Yields simple mass spectra with minimal fragmentation and molecular ions observed. Highly sensitive; capable of detecting gaseous compounds at the pptv level, in a few minutes	Selection of primary ion is important to minimize fragmentation when analyzing neutral gases. Complex gas mixtures (e.g., emission from furnaces, motors) require switching between several primary ions, adding analysis time. Differential pumping requires bulky pumping systems	Dolch et al. (2012a), Dolch et al. (2012b)
	PTR-MS	Portable, high throughput with no previous sample preparation or compound separation. User friendly, less expensive than SIFT-MS. Analyte quantitation is possible at pptv levels. Can complement GC-MS methods	Higher collision energies result in fragmentation and inconsistent product ion formation. The availability of H_3_O^+^ reagent ions only, limits the detection of light hydrocarbons and certain halogenated species	Hansel et al. (1995)
	SESI-MS	Very efficient ionization at atmospheric pressure yields high sensitivity at pptv level. Minimal fragmentation with the molecular ion observed. Easy to interface with commercial ESI MS systems. Suitable for complex biological samples	Vulnerable to matrix-effects causing ion suppression. Not suitable for quantitation. Expensive instrumentation. A nanoflow technique that may result in long sample analysis times	Wüthrich and Giannoukos (2024)
	TOF-MS	A HRMS technique with up to 48 m flight path and 200,000 FWHM mass resolution, at ppb level, across all acquisition speeds. No mass discrimination. Full mass spectra acquired in milliseconds. Can be interfaced with LC, GC.	Duty cycle limited between 1% and 30%. Sensitivity drops with increasing mass resolution. Flight tube sensitive to temperature fluctuation that affects mass accuracy. Not ideal for quantitation	Cooper-Shepherd et al. (2023)
	Orbitrap-MS	Most current HRMS technique with up to 240,000 FWHM mass resolution at *m/z* 400 with sub-ppm accuracy. Internal lock mass calibration with <1 ppm drift over 24 h. High sensitivity. Can be interfaced with off-line techniques	Space charging may limit the dynamic range and sensitivity of low abundance ions. Slow polarity switching in certain models. Increasing scan rates reduce resolution	Zubarev and Makarov (2013)
Off-line	GC-MS	Robust, user-friendly, affordable. High chromatographic resolution with unit mass resolution. Wide linear range. Suitable for quantitation of small molecules in liquid or gaseous samples in diverse matrices. EI detects analytes that are difficult to ionize	Analytes must be easily volatilized. Molecular ion not observed. In convoluted mass spectra, analyte identification may be compromised. Not ideal for isobars and untargeted analysis. Ion source may require frequent cleaning	Lubes and Goodarzi (2017)
	GC-MS/MS	Most sensitive and selective technique for quantification of targeted analytes in complex matrices. Wide linear analytical range; suitable for analytical measurements at sub ppb levels	Upstream sample preparation necessary. Low mass resolution. More expensive than GC-MS. Larger space requirements. Room ventilation may be necessary due to increased heat generation	Tabbal et al. (2022a), Tabbal et al. (2022b)
	GC-TOF-MS	High chromatographic resolution combined with high mass resolution. Simultaneous detection of all ions with accurate mass. Information-rich data acquisition, suitable for complex samples with no loss of data	Mass resolution range is between 30,0000 and 50,000 FWHM with 1–2 ppm mass accuracy. Frequent calibration is necessary. Highly concentrated samples affect accurate mass determinations	Chernushevich et al. (2017)
	GC-Orbitrap MS	Unparalleled mass resolution. Targeted (quantitative) and untargeted (qualitative) analysis, simultaneously. Excellent sensitivity. Compact design. Applications in proteomics, metabolomics, diagnostics, environmental, food, etc., analyses	Limited charge capacity can overfill Orbitrap, reducing mass accuracy. Detection may be affected by ion stability due to conditions in ion injection slit, electrodynamic squeezing, rotational amplitudes, axial oscillation, etc. Expensive instrumentation	Hu et al. (2005), Belarbi et al. (2021)
	GCxGC-HR TOF-MS	Very high peak capacity. Unmatched chromatographic resolution coupled with high mass resolution. FFP design combined with EFP technology improves mass resolution and duty cycle, significantly. Represent the most current GC/MS technology for VOC analysis	Upstream sample preparation is necessary. Very long sample analysis times. Multiple decoding algorithms needed for complex mixtures. Large footprint. Expensive technology	Willis et al. (2021)

^*^For abbreviations, refer to Supplementary Material.

On-line platforms offer benefits in clinical laboratories due to their quick analysis times, lack of sample preparation, user-friendly software interfaces, and portability to point-of-care (Majchrzak et al., 2018; Gould et al., 2021). Quantitation is possible for targeted analytes with prior calibration of the machine using standards (Španěl et al., 1999; Smith and Španěl, 2005; Fernández Maestre, 2012), while non-target analytes are eliminated from the measurement. SIFT-MS instruments provide the flexibility to choose from various precursor ions, including O_2_^+^, H_3_O^+^, and NO^+^, which allows users to select the primary ionization agents most suitable for the analyzed matrix. In contrast, PTR-MS employs H_3_O^+^ as its sole ionizing agent, limiting its versatility but yielding less fragmentation (Smith and Španěl, 2005). On-line techniques are less ideal when conducting untargeted analysis of complex samples where overlapping *m/z* values can limit analyte identification (Kohl et al., 2013; Smith et al., 2014; Lubes and Goodarzi, 2017). Mass resolution can be improved using HRMS systems that reduce the error between accurate mass spectra and predicted mass listed in regulatory-compliant repositories, such as the National Institute of Standards and Technology (NIST) (Ausloos, et al., 1999), Wiley (McLafferty and Sttauffer, 1989), Fiehn (Kind et al., 2009), Golm (Kopka et al., 2004), or other reference libraries. Such repositories utilize information-rich databases and powerful search functions, such as vocBinBase (Skogerson et al., 2011), BinVestigate (Lai et al., 2018) with deconvolution and annotation tools, including MS-DIAL and MS-FINDER (Tsugawa et al., 2015; Tsugawa et al., 2016; Lai et al., 2018), as well as advanced database queries (Kind and Fiehn, 2006). For example, the Wiley Registry/NIST Mass Spectral Library was used to identify various metabolites including terpenoids, saponins, flavonoids, and alkaloids produced by six *Nigella sativa* (black cumin) species (Farag et al., 2014). In a separate integrative study, changes in gene expression and terpenoid production were used to annotate genes and pathways responsible for berry maturation processes (Wang et al., 2017). Despite this potential for multi-omics integration, in the absence of analyte separation even the most accurate mass spectral libraries are confounded by overlapping *m/z* values (Bruderer et al., 2020; Kaeslin et al., 2021).

Off-line platforms, although more labor intensive to operate, offer more detailed chromatographic separations supporting analyte identification, quantification, and structural elucidation (Ahmed et al., 2017; Gould et al., 2021). While development and validation of GC-MS/MS methods requires investment of time and expertise, they remain optimal for profiling VOCs across diverse matrices (Tabbal et al., 2022a; Tabbal et al., 2022b; Berkane et al., 2023). In the analysis of complex samples containing potentially hundreds of analytes, GCxGC-HRMS technology presents several advantages in resolution and analyte identification. The GCxGC module enhances chromatographic peak separation, potentially achieving baseline resolution, and the in-line HRMS system further improves selective spectral identification through high resolution mass measurement of the separated analytes. While low-resolution MS (LRMS) instruments typically measure *m/z* ratios to one decimal place, which suffices for targeted quantification, untargeted analyses require HRMS (Rey-Stolle et al., 2022). HRMS ensures measurement of unknowns with accurate mass where, for example, compounds with nominal mass *m/z* 28.0 have four possible chemical formulas, as shown in Table 3. LRMS is incapable of distinguishing between these possibilities. HRMS compares the measured accurate mass spectra with predicted mass listed in regulatory-compliant repositories and the analyte with the lowest mass error is selected as the most likely hit; in this case *m/z* 28.00562 (Table 3). The chromatographic separation power and accurate mass measurement of GCxGC-HRMS places these platforms at the forefront of VOC detection with the best analyte separation and accurate mass identification (Weggler et al., 2019; Willis et al., 2021; Cooper-Shepherd et al., 2023; LECO Corporation White paper, 2021).

**TABLE 3:** Determination of compounds, *m/z* 28.0 with accurate mass and the possible chemical formulas.

Element	Atomic mass unit	Chemical formula	Nominal mass	Theoretical exact mass	Measured accurate mass	Mass error
		*m/z*	*m/z*	*m/z*	*m/z*	ppm
H	1.00783	N_2_	28.0	28.00559	28.00562	1.07
C	12.00000	CO	28.0	27.99436	27.99429	−2.54
N	14.00307	CH_2_N	28.0	28.01818	28.01827	3.03
O	15.99491	C_2_H_4_	28.0	28.03077	28.03089	4.28
electron	0.00055					

^*^ $Mass\;error=\frac{{mass}_{measured}-{mass}_{theoretical}}{{mass}_{measured}}*10^{6}\;\left[ppm\right]$ .

Living cells and cell systems produce diverse VOC profiles that can be differentially detected using on-line and off-line methods. For example, VOCs produced by plants include terpenoids, phenylpropanoids, benzenoids, and fatty acid derivatives (Picazo-Aragonés et al., 2020; Liu et al., 2023); VOCs detected in blood include alcohols, aldehydes, acids, acetone, hydrogen sulfide, methanethiol, dimethyl sulfide, dimethyldisulfide, trimethylamine, indole, aminoacetophenone (Allardyce et al., 2006a; Allardyce et al., 2006b); VOCs detected in breath include hydrocarbons, alcohols, ketones, aldehydes, carboxylic acids, esters, isoprenoids, furan, nitrogen- and sulfur-containing compounds, aromatics, cyclic hydrocarbons (Issitt et al., 2022; Moura et al., 2023) (https://neomeditec.com/VOCdatabase/); VOCs produced by microorganisms include hydrocarbons, alcohols, aldehydes, acids, ketones, esters, aromatics, phenols, nitrogen- and sulfur-containing compounds (Ratiu et al., 2017); VOCs detected in food include alcohols, aldehydes, acids, esters, terpenes, furans, and pyrazines (Starowicz, 2021); and VOCs detected in environmental samples include alkanes, alkenes, alkynes, alcohols, aldehydes, aromatic compounds, ketones, esters, ethers, haloalkanes, nitriles, organic acids, and acrylamide (Lü et al., 2022). While many of these compound classes are shared between sources, more granular analysis reveals a complex array of molecular forms with potential to serve specific signaling or regulatory roles.

**FIGURE 3:**
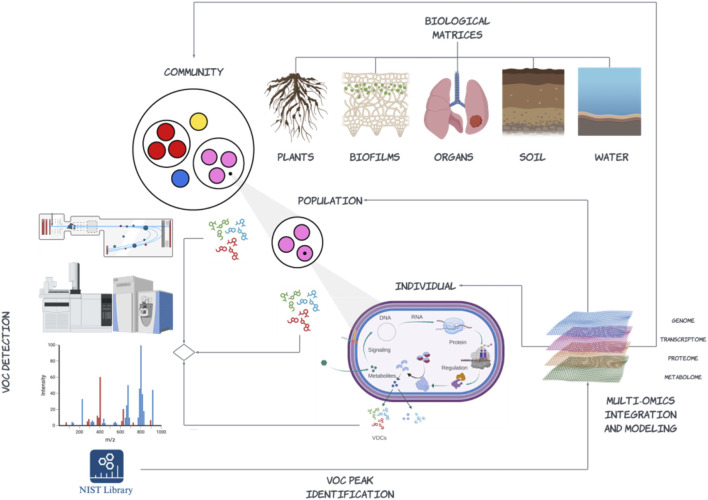
An emerging paradigm for volatilome research integrating multi-omic data (DNA, RNA, protein, and metabolites) spanning different levels of biological organization to resolve volatile organic compound (VOC) profiles emerging from biological matrices sourced from natural or engineered environments including our own bodies. This paradigm includes enrichment and isolation methods to more closely link specific VOCs with specific individuals or populations of microorganisms as well as community-level analysis that define emergent patterns of VOC production and signaling among and between different taxonomic lineages. The development of more extensive regulatory-compliant repositories, e.g., Wiley Registry/National Institute of Standards and Technology (NIST) Mass Spectral Library compound reference libraries and integrative methods linking identified VOCs onto the background metabolic network at scale remain ongoing challenges (Created with BioRender.com).

In this regard there is increasing interest in VOC profiling to develop new metrics that improve understanding of microbial interactions and trait-based contributions to functions and services in natural and engineered environments including our own bodies (Figure 3). For example, microbial VOCs are increasingly being linked back to antimicrobial properties and plant host interactions including defense mechanisms and root growth (Weisskopf et al., 2021; Razo-Belmán et al., 2023; Schmidt et al., 2023). From a diagnostic or biotechnology innovation perspective this not only applies to the detection of VOCs involved in community-level interactions and host associations, but to development of non-invasive point of care diagnostics in health and disease. For example, lung cancer breath analysis has become a promising method of screening, with ∼500 exhaled compounds currently associated with lung cancer status (Schmidt et al., 2023). Identifying correlations between lung microbiome and VOCs in relation to lung cancer status is an active area of research that requires increased throughput, mass resolution and methods standardization. These requirements also represent common challenges to scaling VOC detection in relation to community level interactions as well as in development of screening paradigms to recover genes or gene cassettes producing VOCs from environmental genomes. Despite these challenges, the trajectory of MS platform innovation and continuous improvement in integrative methods of data analysis and statistical modeling provides an exciting opportunity for researchers to ask fundamental questions with real world implications.

## 4 Scope statement

Volatile organic compounds (VOCs) are gas-phase small molecules released from biotic and abiotic matrices into the environment. Because they are volatile, VOCs are typically not detected during conventional metabolite analysis and require specific profiling methods and platforms to measure. One of the most popular platforms is gas chromatography-mass spectrometry that is commonly used for detecting VOCs and can be automated with headspace solid-phase microextraction, etc., methods. The study of VOCs in relation to other forms of biological information, e.g., DNA, RNA, protein, and other metabolites encompasses volatilome research. Emerging lines of evidence suggest that the volatilome plays an integral role in signaling and metabolite exchange within natural and engineered environments including our own bodies where the interplay between microorganisms and host cells defines a complex adaptive network. In this review we trace the evolution of mass spectrometry platforms used in the detection of VOCs in relation to different biological matrices and provide contemporary insight into how VOC profiling is becoming increasingly used to develop non-invasive diagnostic tests across a range of application areas in health, industry, and the environment. Since this review examines VOC analysis using various mass spectrometry platforms with a multi-omics approach, it will fit well in this Research Topic.
