# Supplementary material for: A matrix-centered view of mass spectrometry platform innovation for volatilome research
Source: Front Mol Biosci. 2024 Oct 30;11:1421330. doi: 10.3389/fmolb.2024.1421330 (PMC11557394; doi:10.3389/fmolb.2024.1421330)
Supplement: Supplementary file 1 [file Table1.docx]

**A matrix-centered view of mass spectrometry platform innovation for volatilome research**

Andras Szeitz^1,2,3^, Annika G. Sutton^4^, Steven J. Hallam^1,2,3,5,6,7,8*^

^1^ Genome Science and Technology Program, University of British Columbia, Vancouver, BC, Canada, V6T 1Z4

^2^ Department of Microbiology & Immunology, University of British Columbia, Vancouver, BC, Canada, V6T 1Z3

^3^ Life Sciences Institute, University of British Columbia, Vancouver, BC, Canada, V6T 1Z3

^4^ School of Biomedical Engineering, University of British Columbia, Vancouver, BC, Canada, V6T 2B9

^5^ Graduate Program in Bioinformatics, University of British Columbia, Vancouver, BC, Canada, V6T 1Z4

^6^ Department of Biochemistry, Chulalongkorn University, Bangkok, Thailand, 10330

^7^ Bradshaw Research Institute for Minerals and Mining (BRIMM), University of British Columbia, Vancouver, BC, Canada, V6T 1Z4

^8^ ECOSCOPE Training Program, University of British Columbia, Vancouver, BC, Canada, V6T 1Z3

^*^To whom correspondence should be addressed:

University of British Columbia, Department of Microbiology and Immunology

2552-2350 Health Sciences Mall, Vancouver, BC, Canada V6T, 1Z3

Office: (604) 827-3420 email: shallam@mail.ubc.ca

**Abbreviations**

| 2DGC | Two-dimensional gas chromatography |
| --- | --- |
| ANOVA | Analysis of variance |
| BTEX | Benzene, toluene, ethylbenzene, xylenes |
| DC | Direct current |
| DHS | Dynamic headspace |
| DI-GC-FID | Direct injection gas chromatography flame ionization detection |
| DMS | Differential mobility spectrometry |
| DNA | Deoxyribonucleic acid |
| DuC | Duty cycle |
| EFP | Encoded frequent pushing |
| EI | Electron impact |
| EPA | Environmental Protection Agency |
| ESI | Electrospray ionization |
| GC | Gas chromatography |
| GCxGC | Comprehensive two-dimensional gas chromatography |
| GCxGC-HRMS | Comprehensive two-dimensional gas chromatography high resolution mass spectrometry |
| GCxGC-HR-MR-TOF-MS FFP | Comprehensive two-dimensional gas chromatography high resolution multi-reflecting time-of-flight mass spectrometry folded flight path |
| GCxGC-HR-TOF-MS | Comprehensive two-dimensional gas chromatography high resolution time-of-flight mass spectrometry |
| GCxGC-TOF-MS | Comprehensive two-dimensional gas chromatography time-of-flight mass spectrometry |
| GC-IMS | Gas chromatography ion mobility spectrometry |
| GC-MS | Gas chromatography mass spectrometry |
| GC-MS/MS | Gas chromatography triple quadrupole mass spectrometry |
| HPPI | High-pressure photon ionization |
| HPPI-TOF-MS | High-pressure photon ionization time-of-flight mass spectrometry |
| HR-MR-TOF-MS FFP | Multi-reflecting time-of-flight mass spectrometry with folded flight path |
| HRMS | High-resolution mass spectrometry |
| HR-TOF-MS | High-resolution time-of-flight mass spectrometry |
| HS | Headspace |
| HS-GC-MS/MS | Headspace gas chromatography triple quadrupole mass spectrometry |
| HS-MCC-GC-IMS | Headspace multicapillary column gas chromatography ion mobility spectrometry |
| HS-SPME-GC-MS/MS | Headspace solid phase microextraction gas chromatography triple quadrupole mass spectrometry |
| IMR-MS | Ion-molecule reaction mass spectrometry |
| IMS | Ion mobility spectrometry |
| IMS-MS | Ion mobility spectrometry mass spectrometry |
| LOD | Limit of detection |
| LRMS | Low-resolution mass spectrometry |
| MALDI-TOF-MS | Matrix-assisted laser desorption ionization time-of-flight mass spectrometry |
| MR-GC | Reconfigurable gas chromatography |
| MRM | Multiple reaction monitoring |
| mRNA | Messenger ribonucleic acid |
| MS1 | First quadrupole |
| MS2 | Second quadrupole |
| mVOC | Microbial volatile organic compound |
| oaTOF-MS | Orthogonal acceleration time-of-flight mass spectrometry |
| PBPK | Physiologically based pharmacokinetic |
| PCA | Principal Component Analysis |
| PTR-HRMS | Proton transfer reaction high-resolution mass spectrometry |
| PTR-MS | Proton transfer reaction mass spectrometry |
| PTR-QqQ-MS | Proton transfer reaction triple-quadrupole mass spectrometry |
| PTR-TOF-MS | Proton transfer reaction time-of-flight mass spectrometry |
| RF | Radio frequency |
| Q-MRT | Quadrupole-multi-reflecting time-of-flight |
| RNA | Ribonucleic acid |
| RNA-seq | RNA-sequencing |
| SESI | Secondary electrospray ionization |
| SESI-HRMS | Secondary electrospray ionization high-resolution mass spectrometry |
| SESI-MS | Secondary electrospray ionization mass spectrometry |
| SHS | Static headspace |
| SIFT | Selected ion flow tube |
| SIFT-MS | Selected ion flow tube mass spectrometry |
| SPME | Solid phase microextraction |
| SPME-GC-FID | Solid phase microextraction gas chromatography flame ionization detection |
| SPME-GCxGC-TOF-MS | Solid phase microextraction comprehensive two-dimensional gas chromatography time-of-flight mass spectrometry |
| SPME-GC-MS/MS | Solid phase microextraction gas chromatography triple quadrupole mass spectrometry |
| SPME-GC-TOF-MS | Solid phase microextraction gas chromatography time-of-flight mass spectrometry |
| MS | Mass spectrometry |
| TD | Thermal desorption |
| TD-GC-FID | Thermal desorption gas chromatography flame ionization detection |
| TD-GCxGC-TOF-MS | Thermal desorption comprehensive two-dimensional gas chromatography time-of-flight mass spectrometry |
| TD-GC-MS | Thermal desorption gas chromatography mass spectrometry |
| TD-GC-MS/MS | Thermal desorption gas chromatography triple quadrupole mass spectrometry |
| TOF | Time-of-flight |
| TOF-MS | Time-of-flight mass spectrometry |
| VFA | Volatile fatty acid |
| VOC | Volatile organic compound |
| WHO | World Health Organization |
